# Fixed point theorems for nonlinear contractive mappings in ordered *b*-metric space with auxiliary function

**DOI:** 10.1186/s13104-020-05273-1

**Published:** 2020-09-22

**Authors:** Namana Seshagiri Rao, Karusala Kalyani, Belay Mitiku

**Affiliations:** 1 Department of Applied Mathematics, School of Applied Natural Sciences, Adama Science and Technology University, Post Box No.1888, Adama, Ethiopia; 2 Department of Mathematics, Vignan’s Foundation for Science, Technology & Research, Vadlamudi, Andhra Pradesh 522213 India

**Keywords:** Ordered *b*-metric space, Rational type generalized contraction mappings, Fixed points, Complete metric space, 46T99, 41A50, 54H25

## Abstract

**Objectives:**

In this paper we present some fixed point theorems for self mappings satisfying generalized $(\phi , \psi )$-weak contraction condition in partially ordered complete *b*-metric spaces. The results presented over here generalize and extend some existing results in the literature. Finally, we illustrate two examples to support our results.

**Result:**

We obtained a unique fixed point of a self mapping satisfying certain contraction condition which is involving an auxiliary function. Also, the results are presented for the existence of a common fixed point and a coincidence point for generalized $(\phi , \psi )$-weak contraction mappings in partially ordered complete *b*-metric space.

## Introduction

Fixed points of mappings satisfying contractive conditions in generalized metric spaces are highly useful in large number of mathematical problems of pure and applied mathematics. First, Ran and Reuings [1] have extended the result in this direction, discussed the existence of fixed points for certain maps in ordered metric space and also presented some applications to matrix linear equations. Afterwords, the result of [1] has been extended by Nieto et al. [2, 3] involving nondecreasing mappings and used their results in obtaining an unique solution of a first order differential equation. At the same time, the results regarded to generalized contractions in ordered spaces were studied by Agarwal et al. [4] and, O’Regan et al. [5]. The concept of coupled fixed points for certain mappings was first introduced by Bhaskar and Lakshmikantham [6] and then applied their results to a periodic boundary value problem in acquiring an unique solution. Thereafter, the concept of coupled coincidence and common fixed point results was first initiated by Lakshmikantham and Ćirić [7], which were the extensions of Bhaskar and Lakshmikantham [6] involving monotone property for a function in ordered metric spaces. More work relevant to coupled fixed point results under different contractive conditions in various spaces can be found from [8–15]. Later Singh et al. [16] obtained a coincidence and common fixed point theorems for Suzuki type hybrid contractions in ordered metric spaces and presented the corresponding applications to the results in their work. Mean while a new type of coincidence and common fixed point theorems with applications was investigated by Singh et al. [17].

*b*-metric space is one of many generalizations to an usual metric, which was first introduced by Bakhtin [18] in his work and then extensively used by Czerwik in [19, 20]. Thereafter, lot of improvements have been done in finding fixed points for single, multi-valued operator in that space, the readers may refer to [21–29].

In this paper, we introduce the class of generalized $(\phi , \psi )$-weak contraction to establish an existence of a fixed point and its uniqueness of a self mapping and common fixed point, coincidence point for two self mappings in ordered complete *b* metric space. Our results generalize Theorem 2.1 and 2.2 of [38] and the Corollaries of [39] and several comparable results in the literature [32, 35–37].

## Mathematical preliminaries

We start this section with the following definitions and results which are frequently used in the main results.

### **Definition 1**

[20] A mapping $d: P \times P \rightarrow [0, +\infty )$, where *P* is a non-empty set is said to be a *b*-metric, if it satisfies the properties given below for any $\nu ,\xi ,\mu ,\in P$ and for some $s \ge 1$,

- $d(\nu ,\xi )=0$ if and if $\nu =\xi$,
- $$d(\nu ,\xi )=d(\xi ,\nu )$$,
- $$d(\nu ,\xi ) \le s \left( d(\nu ,\mu )+d(\mu ,\xi )\right)$$.

And then (*P*, *d*, *s*) is known as a *b*-metric space.

### **Definition 2**

[20] Let (*P*, *d*, *s*) be a *b*-metric space. Then

1. a sequence $\{\nu _n\}$ is said to converge to $\nu$ if $\lim \limits _{n \rightarrow +\infty }d(\nu _n,\nu )=0$ and written as $\lim \limits _{n \rightarrow +\infty }\nu _n=\nu$.
2. $\{\nu _n\}$ is said to be a Cauchy sequence in *P*, if $\lim \limits _{n,m \rightarrow +\infty }d(\nu _n,\nu _m)=0$.
3. (*P*, *d*, *s*) is said to be complete if every Cauchy sequence in *P* is convergent.

### **Definition 3**

A metric *d* on *P* together with a partially ordered relation $\le$ is called a partially ordered metric space. It is denoted by $(P,d,\le )$.

### **Definition 4**

If the metric *d* is complete then $(P,d,\le )$ is called a complete partially ordered metric space.

### Definition 5

Let $(P,\le )$ be a partially ordered set. A map $S:P \rightarrow P$ is said to be monotone nondecreasing, if $S(\nu )\le S(\xi )$ for all $\nu ,\xi \in P$ with $\nu \le \xi$.

### **Definition 6**

A point $\nu \in A$, where $A\ne \emptyset$, a subset of (*P*, *d*) is called a common fixed (coincidence ) point for two self-mappings *f* and *S*, if $\nu =f\nu =S\nu ~(f\nu =S\nu )$.

### **Definition 7**

Two self maps *f* and *S* defined over a subset *A* of (*P*, *d*) are called commuting, if $fS\nu =Sf\nu$, for all $\nu \in A$.

### **Definition 8**

Two self maps *f* and *S* defined over $A\ne \emptyset$, a subset of (*P*, *d*) are called compatible, if any sequence $\{\nu _n\}$ with $\lim \limits _{n \rightarrow +\infty }f\nu _n= \lim \limits _{n \rightarrow +\infty }S\nu _n =\mu ,~ \text {for}~ \mu \in A$ then $\lim \limits _{n \rightarrow +\infty } d(Sf\nu _n,fS\nu _n) =0$.

### **Definition 9**

A pair of self mappings (*f*, *S*) on $A\ne \emptyset$, a subset of *P* is called weakly compatible, if $Sf\nu =fS\nu$, when $S\nu =f\nu$ for some $\nu \in A$.

### **Definition 10**

Let *f* and *S* be two self-mappings over $(P,\le )$. Then *S* is called monotone *f* nondecreasing, if

$$\begin{aligned} f\nu \le f\xi \Rightarrow S\nu \le S\xi ,~\text {for any} ~\nu ,\xi \in P. \end{aligned}$$

### **Definition 11**

If very two elements of $A\ne \emptyset$, a sub set of $(P,\le )$ are comparable then *A* is called well ordered set.

The concept of acquiring fixed points in metric space using control functions was initiated by Khan et al. [30].

### **Definition 12**

[30] A self map $\phi$ defined on $[0, +\infty )$ is said to be an altering distance function, if $\phi$ is continuous and monotone increasing with $\phi (t)=0$ iff $t=0$.

**Note 1**

(1): *Let us denote the set of all altering distance functions on* $[0, +\infty )$*by* $\Phi$.
(2): *Similarly*, $\Psi$*denoted by the set of lower semi-continuous functions on* $[0, +\infty )$*with* $\psi (t)=0$*iff* $t=0$.

### **Lemma 1**

[31] *Let* *P* *be a non-empty set and* $f: P \rightarrow P$*be a mapping*. *Then there exists a subset* *E* *of* *P* *such that* $fE=fP$*and* $f:E \rightarrow P$*is one-to-one*.

In 1975, Dass and Gupta [32] proved the following fixed point result in a complete metric space.

### **Theorem 1**

[32] *Suppose* (*P*, *d*) *is a complete metric space. Let* $S: P \rightarrow P$*be a mapping such that there exist* $\alpha , \beta \in [0,1)$*with* $\alpha + \beta <1$*satisfying*

1 $$\begin{aligned} d(S\nu ,S\xi )\le \alpha \frac{d(\xi ,S\xi ) \left[ 1+d(\nu ,S\nu )\right] }{1+d(\nu ,\xi )} +\beta d(\nu ,\xi ) \end{aligned}$$

*for any distinct* $\nu , \xi \in P$. *Then* *S* *has a unique fixed point in* *P*.

The generalization of above result in partially ordered metric space was obtained by Cabrera et al. [33] in 2013. Later Chandok et al. [34] generalized the result of [33] by use of control functions in the space.

Again, Theorem 1 was generalized by Jaggi [35] in 1977 and proved the following

### **Theorem 2**

[35] *Suppose* (*P*, *d*) *is a complete metric space. A self map* *S* *on* *P* *such that*

2 $$\begin{aligned} d(S\nu ,S\xi )\le \alpha \frac{d(\xi ,S\xi )~d(\nu ,S\nu )}{d(\nu ,\xi )} +\beta d(\nu ,\xi ) \end{aligned}$$

*for all* $\nu , \xi \in P$*with* $\nu \ne \xi$, *where* $\alpha , \beta \in [0,1)$*with* $\alpha + \beta <1$. *Then* *S* *has a unique fixed point in* *P*.

This result was again proved by Harjani et al. [36] in a complete metric space endowed with partial order relation. Later the result of [36] was generalized by Luong et al. [37] involving altering distance functions which satisfies a weak contractive condition of, rational type auxiliary functions in ordered metric space. Thereafter, the result [37] was generalized and extended by Chandok et al. [38] in 2013 and obtained coupled, common fixed point results for weak contractive mapping in partially ordered metric space. These results were again generalized by Hieu et al. [39] in partially order *b*-metric space by involving altering distance functions.

## Main text

To begin this section with the following theorem.

### **Theorem 3**

*Let* $(P,d,s,\le )$*be a complete partially ordered* *b*-*metric space with parameter* $s \ge 1$. *Let* $S:P \rightarrow P$*be a continuous, nondecreasing mapping with regards to* $\le$*such that there exists* $\nu _0 \in P$*with* $\nu _0 \le S\nu _0$. *Suppose that*

3 $$\begin{aligned} \phi (sd(S\nu ,S\xi ))\le \phi (M(\nu ,\xi ))-\psi (M(\nu ,\xi )) \end{aligned}$$

*where* $\phi \in \Phi , \psi \in \Psi$, *for any* $\nu ,\xi \in P$*with* $\nu \le \xi$*and*

4 $$\begin{aligned} M(\nu ,\xi )=\max \left\{ \frac{d(\xi ,S\xi ) \left[ 1+d(\nu ,S\nu )\right] }{1+d(\nu ,\xi )},\frac{d(\xi ,S\nu ) \left[ 1+d(\nu ,S\xi )\right] }{1+d(\nu ,\xi )}, d(\nu ,\xi )\right\} . \end{aligned}$$

*Then* *S* *has a fixed point in* *P*.

### *Proof*

If for some $\nu _0 \in P$ such that $S\nu _0=\nu _0$, then the proof is finished. Assume that $\nu _0 < S\nu _0$, then construct a sequence $\{\nu _n\} \subset P$ by $\nu _{n+1}=S\nu _n$, for $n\ge 0$. But *S* is nondecreasing then, we get the following by using mathematical induction

5 $$\begin{aligned} \nu _0 < S\nu _0=\nu _1\le S\nu _1=\nu _2\le .............\le S\nu _{n-1}=\nu _n \le S\nu _n=\nu _{n+1}\le ........ \end{aligned}$$

If for some $n_0\in {\mathbb {N}}$ such that $\nu _{n_0}=\nu _{n_0+1}$ then from (5), $\nu _{n_0}$ is a fixed point of *S* and we have nothing to prove. Suppose that $\nu _n \ne \nu _{n+1}$, i.e., $d(\nu _n, \nu _{n+1})>0$, for all $n \ge 1$. Since $\nu _n>\nu _{n-1}$, for any $n \ge 1$ and then by (3), we have

6 $$\begin{aligned} \begin{aligned} \phi (d(\nu _n,\nu _{n+1}))&= \phi (d(S\nu _{n-1},S\nu _n))\le \phi (sd(S\nu _{n-1},S\nu _n))\\ {}&\le \phi (M(\nu _{n-1},\nu _n))-\psi (M(\nu _{n-1},\nu _n)), \end{aligned} \end{aligned}$$

where

7 $$\begin{aligned} \begin{aligned} M(\nu _{n-1}, \nu _n)&=\max \left\{ \frac{d(\nu _n,S\nu _n) \left[ 1+d(\nu _{n-1},S\nu _{n-1})\right] }{1+d(\nu _{n-1},\nu _n)}, \frac{d(\nu _n,S\nu _{n-1})\left[ 1+d(\nu _{n-1},S\nu _n)\right] }{1+d(\nu _{n-1},\nu _n)},\right. \\&\quad \left. d(\nu _{n-1},\nu _n)\right\} \\&= \max \{d(\nu _n,\nu _{n+1}),0, d(\nu _{n-1},\nu _n)\}\\&= \max \{d(\nu _n,\nu _{n+1}),d(\nu _{n-1},\nu _n)\} \end{aligned} \end{aligned}$$

which implies that

8 $$\begin{aligned} \phi (d(\nu _n,\nu _{n+1}))\le \phi (\max \{d(\nu _n,\nu _{n+1}),d(\nu _{n-1},\nu _n)\})-\psi (\max \{d(\nu _n,\nu _{n+1}),d(\nu _{n-1},\nu _n)\}). \end{aligned}$$

If $\max \{d(\nu _n,\nu _{n+1}), d(\nu _{n-1},\nu _n)\}= d(\nu _n,\nu _{n+1})$ for some $n \ge 1$, then from (8), we get

9 $$\begin{aligned} \phi (d(\nu _n,\nu _{n+1})) \le \phi (d(\nu _n,\nu _{n+1}))-\psi (d(\nu _n,\nu _{n+1}))<\phi (d(\nu _n,\nu _{n+1})), \end{aligned}$$

which is a contradiction under (9). Thus, $\max \{d(\nu _n,\nu _{n+1}), d(\nu _{n-1},\nu _n)\}= d(\nu _{n-1},\nu _n)$ for $n \ge 1$ and hence from (8) again we have

10 $$\begin{aligned} \phi (d(\nu _n,\nu _{n+1})) \le \phi (d(\nu _n,\nu _{n-1}))-\psi (d(\nu _n,\nu _{n-1}))<\phi (d(\nu _n,\nu _{n-1})). \end{aligned}$$

Therefore, the sequence $\{d(\nu _n,\nu _{n-1})\}$ for $n \ge 1$ is a monotone non-increasing and bounded. From a result, we have

11 $$\begin{aligned} \lim \limits _{n \rightarrow +\infty }d(\nu _n,\nu _{n-1})=\rho \ge 0. \end{aligned}$$

Now, taking the upper limit on both sides of (6), we obtain

12 $$\begin{aligned} \phi (\rho ) \le \phi (\rho )-\lim \limits _{n \rightarrow +\infty } \inf \psi (d(\nu _n,\nu _{n-1}))\le \phi (\rho )-\psi (\rho )<\phi (\rho ) \end{aligned}$$

which is a contradiction under (12). Thus, $\rho =0$. Hence, $d(\nu _n,\nu _{n-1})\rightarrow 0$ as $n \rightarrow +\infty$.

Next, we prove that $\{\nu _n\}$ is a Cauchy sequence in *P*. Assume opposite that $\{\nu _n\}$ is not a Cauchy sequence. Then for some $\epsilon >0$, we can get two subsequences $\{\nu _{m_j}\}$ and $\{\nu _{n_j}\}$ of $\{\nu _n\}$, where $n_j$ is the smallest index such that

13 $$\begin{aligned} n_j>m_j>j,~~~~~ d(\nu _{m_j},\nu _{n_j})\ge \epsilon \end{aligned}$$

and

14 $$\begin{aligned} d(\nu _{m_j},\nu _{{n_j}-1}) < \epsilon . \end{aligned}$$

Applying triangular inequality in (13), we get

15 $$\begin{aligned} \begin{aligned} \epsilon&\le d(\nu _{m_j},\nu _{n_j})\le s d(\nu _{m_j},\nu _{{n_j}-1})+sd(\nu _{n_j-1},\nu _{n_j})\\&\le s^2d(\nu _{m_j},\nu _{{m_j}-1})+s^2d(\nu _{{m_j}-1}, \nu _{{n_j}-1})+sd(\nu _{{n_j}-1},\nu _{n_j}) \end{aligned} \end{aligned}$$

Furthermore,

16 $$\begin{aligned} \begin{aligned} d(\nu _{{m_j}-1},\nu _{{n_j}-1})\le s d(\nu _{{m_j}-1},\nu _{m_j})+sd(\nu _{m_j},\nu _{{n_j}-1}) \le s d(\nu _{{m_j}-1},\nu _{{m_j}})+s \epsilon \end{aligned} \end{aligned}$$

Letting $j \rightarrow +\infty$ in Eqs. (15), (16) and combining together, we obtain the following inequality

17 $$\begin{aligned} \frac{\epsilon }{s^2}\le \lim \limits _{j \rightarrow +\infty } \sup d(\nu _{{m_j}-1},\nu _{{n_j}-1}) \le s \epsilon . \end{aligned}$$

Similarly, we can get the following inequalities by using triangular inequality

18 $$\begin{aligned} \frac{\epsilon }{s^2}\le \lim \limits _{j \rightarrow +\infty } \inf d(\nu _{{m_j}-1},\nu _{{n_j}-1}) \le s \epsilon , \end{aligned}$$

and

19 $$\begin{aligned} \frac{\epsilon }{s}\le \lim \limits _{j \rightarrow +\infty } \sup d(\nu _{{m_j}-1},\nu _{n_j}) \le s \epsilon ^2. \end{aligned}$$

Let

20 $$\begin{aligned} \begin{aligned} M(\nu _{{m_j}-1}, \nu _{{n_j}-1})&=\max \left\{ \frac{d(\nu _{{n_j}-1},S\nu _{{n_j}-1}) \left[ 1+d(\nu _{{m_j}-1},S\nu _{{m_j}-1})\right] }{1+d(\nu _{{m_j}-1}, \nu _{{n_j}-1})},\right. \\&\quad \left. \frac{d(\nu _{{n_j}-1},S\nu _{{m_j}-1}) ~\left[ 1+d(\nu _{{m_j}-1},S\nu _{{n_j}-1})\right] }{1+d(\nu _{{m_j}-1}, \nu _{{n_j}-1})}, d(\nu _{{m_j}-1},\nu _{{n_j}-1})\right\} \\&=\max \left\{ \frac{d(\nu _{{n_j}-1},\nu _{n_j})\left[ 1+d(\nu _{{m_j}-1},\nu _{m_j}) \right] }{1+d(\nu _{{m_j}-1}, \nu _{{n_j}-1}) },\right. \\&\quad \left. \frac{d(\nu _{{n_j}-1},\nu _{m_j})\left[ 1+d(\nu _{{m_j}-1},\nu _{n_j})\right] }{1+d(\nu _{{m_j}-1},\nu _{{n_j}-1})}, d(\nu _{{m_j}-1}, \nu _{{n_j}-1})\right\} . \end{aligned} \end{aligned}$$

From (20), we obtain the following inequalities

21 $$\begin{aligned} \frac{\epsilon }{s^2}\le \lim \limits _{j \rightarrow +\infty }\sup M (\nu _{{m_j}-1}, \nu _{{n_j}-1}) \le s \epsilon . \end{aligned}$$

and

22 $$\begin{aligned} \frac{\epsilon }{s^2}\le \lim \limits _{j \rightarrow +\infty } \inf M(\nu _{{m_j}-1}, \nu _{{n_j}-1}) \le s \epsilon . \end{aligned}$$

Form (5), we have $\nu _{{m_j}-1}<\nu _{{n_j}-1}$, then

23 $$\begin{aligned} \phi (sd(\nu _{m_j},\nu _{n_j}))=\phi (sd(S\nu _{{m_j}-1},S\nu _{{n_j}-1})) \le \phi (M(\nu _{{m_j}-1},\nu _{{n_j}-1}))- \psi (M(\nu _{{m_j}-1},\nu _{{n_j}-1})). \end{aligned}$$

Now, letting $j \rightarrow +\infty$ and use of (21), (22), we obtain

24 $$\begin{aligned} \begin{aligned} \phi (s \epsilon )&\le \phi (s \lim \limits _{j \rightarrow +\infty } d(\nu _{m_j}, \nu _{n_j}))\\&\le \phi ( \lim \limits _{j \rightarrow +\infty } \sup M (\nu _{{m_j}-1}, \nu _{{n_j}-1}))- \lim \limits _{j \rightarrow +\infty }\inf \psi ( M (\nu _{{m_j}-1}, \nu _{{n_j}-1}))\\&\le \phi (s \epsilon )- \psi (\lim \limits _{j \rightarrow +\infty }\inf M (\nu _{{m_j}-1}, \nu _{{n_j}-1}))\\&<\phi (s \epsilon ) \end{aligned} \end{aligned}$$

this is a contradiction under (24). Hence, $\{\nu _n\}$ is a Cauchy sequence and converges to some $\mu \in P$ as *P* is complete. Also, the continuity of *S* implies that

25 $$\begin{aligned} S\mu =S(\lim \limits _{n\rightarrow +\infty }\nu _n)=\lim \limits _{n\rightarrow +\infty }S\nu _n=\lim \limits _{n\rightarrow +\infty }\nu _{n+1}=\mu . \end{aligned}$$

Therefore, $\mu$ is a fixed point of *S* in *P*. $\square$

By weakening the continuity property of a map *S* in Theorem 3, we have the following result.

### **Theorem 4**

*In Theorem* 3, *if* *P* *has a property that, the sequence* $\{\nu _n\}$*is a nondecreasing such that* $\nu _n\rightarrow v$, *implies that* $\nu _n \le v$, *for all* $n \in {\mathbb {N}}$, *i.e*., $v=\sup \nu _n$*then a non continuous map* *S* *has a fixed point in* *P*.

### *Proof*

From Theorem 3, we take the same sequence $\{\nu _n\}$ in *P* such that $\nu _0 \le \nu _1\le \nu _2\le \nu _3\le ........\le \nu _n\le \nu _{n+1}\le ..........$, i.e., the sequence $\{\nu _n\}$ is nondecreasing and converges to some *v* in *P*. Thus, from the hypotheses we have $\nu _n \le v$, for any $n \in {\mathbb {N}}$, implies that $v=\sup \nu _n$.

Next, we prove that *v* is a fixed point of *S* in *P*, that is $Sv=v$. Suppose $Sv\ne v$, that is $d(Sv,v)\ne 0$. Let

26 $$\begin{aligned} \begin{aligned} M(\nu _n, v)&=\max \left\{ \frac{d(v,Sv) \left[ 1+d(\nu _n,S\nu _n)\right] }{1+d(\nu _n, v)}, \frac{d(v,S\nu _n)\left[ 1+d(\nu _n,Sv)\right] }{1+d(\nu _n, v)}, d(\nu _n, v)\right\} \\&=\max \left\{ \frac{d(v,Sv) \left[ 1+d(\nu _n,\nu _{n+1})\right] }{1+d(\nu _n,v)}, \frac{d(v,\nu _{n+1})\left[ 1+d(\nu _n,Sv)\right] }{1+d(\nu _n,v)}, d(\nu _n,v)\right\} \end{aligned} \end{aligned}$$

Letting $n\rightarrow +\infty$ and from $\lim \limits _{n\rightarrow +\infty }\nu _n=v$, we get

27 $$\begin{aligned} \lim \limits _{n \rightarrow +\infty }M(\nu _n, v)= \max \{d(v,Sv),0,0\}=d(v,Sv). \end{aligned}$$

We know that $\nu _n \le v$, for all *n* then from contraction condition (3), we get

28 $$\begin{aligned} \phi (d(\nu _{n+1}, Sv))=\phi (d(S\nu _n, Sv)\le \phi (s d(S\nu _n, Sv)\le \phi (M(\nu _n, v))-\psi (M(\nu _n, v)) \end{aligned}$$

Letting $n \rightarrow +\infty$ and use of (27), we get

29 $$\begin{aligned} \phi (d(v,Sv)) \le \phi (d(v,Sv))-\psi (d(v,Sv))< \phi (d(v,Sv)) \end{aligned}$$

which is wrong under (29). Thus, $Sv=v$, that is *S* has a fixed point *v* in *P*. $\square$

The uniqueness of, an existing fixed point in Theorems 3 and 4 can get, if *P* has the following property:

For any $$\nu$$, $$\xi$$$$\in P$$, there exists $$v \in P$$ such that $$v \le \nu$$ and $$v \le \xi$$.

### **Theorem 5**

*If* *P* *satisfies the above mentioned condition in Theorem *3 (*or Theorem* 4) *then* *S* *has a unique fixed point*.

### *Proof*

From Theorem 3 (or Theorem 4), we conclude that *S* has a nonempty set of fixed points. Suppose that $\nu ^*$ and $\xi ^*$ be two fixed points of *S* then, we claim that $\nu ^*=\xi ^*$. Suppose that $\nu ^*\ne \xi ^*$, then from the hypotheses we have

30 $$\begin{aligned} \begin{aligned} \phi (d(S\nu ^*, S\xi ^*))&\le \phi (sd(S\nu ^*, S\xi ^*)) \le \phi (M(\nu ^*, \xi ^*))-\psi (M(\nu ^*, \xi ^*)) \end{aligned} \end{aligned}$$

where

31 $$\begin{aligned} \begin{aligned} M(\nu ^*, \xi ^*)&=\max \left\{ \frac{d(\xi ^*,S\xi ^*) \left[ 1+d(\nu ^*,S\nu ^*)\right] }{1+d(\nu ^*,\xi ^*)}, \frac{d(\xi ^*,S\nu ^*) \left[ 1+d(\nu ^*,S\xi ^*)\right] }{1+d(\nu ^*,\xi ^*)}, d(\nu ^*,\xi ^*)\right\} \\&= \max \left\{ \frac{d(\xi ^*,\xi ^*) \left[ 1+d(\nu ^*,\nu ^*)\right] }{1+d(\nu ^*,\xi ^*)},\frac{d(\xi ^*,\nu ^*) \left[ 1+d(\nu ^*,\xi ^*)\right] }{1+d(\nu ^*,\xi ^*)}, d(\nu ^*,\xi ^*)\right\} \\&= \max \{0,d(\xi ^*,\nu ^*), d(\nu ^*,\xi ^*) \}\\&=d(\nu ^*,\xi ^*). \end{aligned} \end{aligned}$$

Then by Eq. (30), we have

32 $$\begin{aligned} \begin{aligned} \phi (d(\nu ^*, \xi ^*))&=\phi (d(S\nu ^*, S\xi ^*)) \le \phi (d(\nu ^*, \xi ^*))-\psi (d(\nu ^*, \xi ^*)) < \phi (d(\nu ^*, \xi ^*)) \end{aligned} \end{aligned}$$

which is a contradiction under (32). Hence, $\nu ^*= \xi ^*$.

The proof is completed. $\square$

Now, we have the results below, which are the generalizations of Theorems-2.1 and 2.2 of [38] and the Corollaries-2.1 and 2.2 of [39] in the space.

### **Corollary 1**

*Let* $(P,d,s,\le )$*be a partially ordered* *b-metric space with a parameter* *s*. *Suppose* $S,f: P \rightarrow P$*are continuous mappings such that*

$$(C_1)$$.
*for some* $\psi \in \Psi$*and* $\phi \in \Phi$*with*
  33 $$\begin{aligned} \phi (sd(S\nu ,S\xi ))\le \phi (M_f(\nu ,\xi ))-\psi (M_f(\nu ,\xi )) \end{aligned}$$
  *for any* $$\nu$$, $$\xi$$$$\in P$$*such that* $$f\nu \le f\xi$$*and*
  34 $$\begin{aligned} M_f(\nu ,\xi )=\max \left\{ \frac{d(f\xi ,S\xi ) \left[ 1+d(f\nu ,S\nu )\right] }{1+d(f\nu ,f\xi )},\frac{d(f\xi ,S\nu ) \left[ 1+d(f\nu ,S\xi )\right] }{1+d(f\nu ,f\xi )}, d(f\nu ,f\xi )\right\} , \end{aligned}$$
$$(C_2)$$
$SP \subset fP$*and* *fP* *is a complete subspace of* *P*,
$$(C_3)$$
*S* *is a monotone* *f*-*non decreasing mapping*,
$$(C_4)$$
*S* *and* *f* *are compatible*.

*If for some* $\nu _0 \in P$*such that* $f\nu _0 \le S\nu _0$, *then* *S* *and* *f* *have a coincidence point in* *P*.

### *Proof*

By using lemma 1, we obtain a complete subspace *fE* of *P*, where $E \subset P$ and *f* is one-to-one self mapping on *P*. By Corollary 2.1 of [39], we have a sequence $\{f\nu _n\} \subset fE$ for some $\nu _0 \in E$ with $f\nu _{n+1}=S\nu _n=g(f\nu _n)$, for $n \ge 0$, where *g* is a self-mapping on *fE* with $g(f\nu )=S\nu$, $\nu \in E$. So, from the hypotheses we have

35 $$\begin{aligned} \phi (sd(g(f\nu ),g(f\xi )))\le \phi (M_f(\nu ,\xi ))-\psi (M_f(\nu ,\xi )) \end{aligned}$$

for all $$\nu$$, $$\xi$$$$\in P$$ with $$f\nu \le f\xi$$ and,

36 $$\begin{aligned} M_f(\nu ,\xi )=\max \left\{ \frac{d(f\xi ,g(f\xi )) \left[ 1+d(f\nu ,g(f\nu ))\right] }{1+d(f\nu ,f\xi )}, \frac{d(f\xi ,g(f\nu ))\left[ 1+d(f\nu ,g(f\xi ))\right] }{1+d(f\nu ,f\xi )}, d(f\nu ,f\xi )\right\} . \end{aligned}$$

From the same argument in Theorem 3, $\{f\nu _n\}$ is a Cauchy sequence and which converges for some $v \in fE$. Thus, by the compatibility of *S* and *f*, we obtain

37 $$\begin{aligned} \lim \limits _{n \rightarrow +\infty }d(f(S\nu _n), S(f\nu _n))=0. \end{aligned}$$

Further, by use of triangular inequality,

38 $$\begin{aligned} d(Sv,fv)\le sd(Sv,S(f\nu _n))+s^2 d(S(f\nu _n), f(S\nu _n))+s^2d(f(S\nu _n), fv). \end{aligned}$$

Finally, we arrive at $d(Sv,fv)=0$ as $n \rightarrow +\infty$ in (38). Therefore, *v* is a coincidence point for *S* and *f* in *P*. $\square$

Replace the condition, weakly compatible instead of (*C*4) in Corollary 1, we obtain the following result.

### **Corollary 2**

*If* *P* *has the property in Corollary* 1*instead of the compatibility for* *S* *and* *f* *that, for any nondecreasing sequence* $\{f\nu _n\}\subset P$*such that* $\lim \limits _{n \rightarrow +\infty } f\nu _n=f\nu$*implies that* $f\nu _n \le f\nu$*for all* $n \in {\mathbb {N}}$, *that is* $f\nu =\sup f\nu _n$. *Then* *S* *and* *f* *have a common fixed point in* *P*, *if for some coincidence point* $\mu$*of* *S* *and* *f* *with* $f\mu \le f(f\mu )$. *Furthermore, the set of common fixed points of* *S* *and* *f* *is well ordered if and only if* *S* *and* *f* *have one and only one common fixed point*.

### *Proof*

It is obvious from Corollary 1 and Theorem 4 that *S* and *f* have a coincidence point in *P*, as $f\mu =g(f\mu )=S\mu$ for some $\mu$ in *P*.

Next, assume that a pair of mappings (*S*, *f*) is weakly compatible and let $\vartheta$ be an element in *P* such that $\vartheta =S\mu =f\mu$. Then, $S\vartheta =S(f\mu )=f(S\mu )=f\vartheta$. Let

39 $$\begin{aligned} \begin{aligned} M(\mu ,\vartheta )&=\max \left\{ \frac{d(f\vartheta ,S\vartheta ) \left[ 1+d(f\mu ,S\mu )\right] }{1+d(f\mu ,f\vartheta )},\frac{d(f\vartheta ,S\mu ) \left[ 1+d(f\mu ,S\vartheta )\right] }{1+d(f\mu ,f\vartheta )}, d(f\mu ,f\vartheta )\right\} \\&=\max \{0,d(S\mu ,S\vartheta )\}\\&=d(S\mu ,S\vartheta ). \end{aligned} \end{aligned}$$

then from contraction condition, we have

40 $$\begin{aligned} \begin{aligned} \phi (d(S\mu ,S\vartheta )) \le \phi (M(\mu ,\vartheta ))-\psi (M(\mu ,\vartheta )) \le \phi (d(S\mu ,S\vartheta ))-\psi (d(S\mu ,S\vartheta )). \end{aligned} \end{aligned}$$

Hence, we get $d(S\mu ,S\vartheta )=0$ by the property of $\psi$. Therefore, $S\vartheta =f\vartheta =\vartheta$.

Eventually, by following Theorem 5, we deduce that *S* and *f* have one and only one common fixed point if and only if the set of common fixed points of *S* and *f* is well ordered. $\square$

We illustrate the usefulness of the obtained results in different cases such as continuity and discontinuity of a metric *d* in a space *P*.

### *Example 1*

Define a metric $d:P \rightarrow P$ as below and $\le$ be an usual order in *P*, where $P=\{1,2,3,4,5\}$

$$\begin{aligned}&d(\nu , \xi )=d(\xi ,\nu )=0, ~\text {if}~ \nu , \xi =1,2,3,4,5 ~\text {and}~ \nu = \xi .\\&d(\nu , \xi )=d(\xi ,\nu )=1, ~if~ \nu ,\xi =1,2,3,4 ~\text {and}~ \nu \ne \xi .\\&d(\nu , \xi )=d(\xi ,\nu )=6, ~if~ \nu =1,2,3 ~\text {and}~ \xi =5.\\&d(\nu , \xi )=d(\xi ,\nu )=12, ~if~ \nu =4 ~\text {and}~ \xi =5. \end{aligned}$$

Define a map $S:P \rightarrow P$ by $S1=S2=S3=S4=1, S5=3$ and let $\phi (t)=\frac{t}{2}$, $\psi (t)=\frac{t}{3}$ for $t \in [0,+\infty )$. Then *S* has a fixed point in *P*.

### *Proof*

It is apparent that, $(P,d,s,\le )$ is a complete partially ordered *b*-metric space for $s=2$. Consider the possible cases for $\nu$, $\xi$ in *P*:

**Case 1** Suppose $\nu , \xi \in \{1,2,3,4\}$ and $\nu <\xi$, thence,

$$\begin{aligned} \phi (2d(S\nu ,S\xi ))=0 \le \phi (M(\nu ,\xi ))-\psi (M(\nu ,\xi )). \end{aligned}$$

**Case 2** Suppose that $\nu \in \{1,2,3,4\}$ and $\xi =5$, then $d(S\nu ,S\xi )=d(1,3)=1$, $M(4,5)=12$ and $M(\nu ,5)=6$, for $\nu \in \{1,2,3\}$. Therefore, we have the following inequality,

$$\begin{aligned} \phi (2d(S\nu ,S\xi )) \le \frac{M(\nu ,\xi )}{6} =\phi (M(\nu ,\xi ))-\psi (M(\nu ,\xi )). \end{aligned}$$

Thus, condition (3) of Theorem 3 holds. Furthermore, the remaining assumptions in Theorem 3 are fulfilled. Hence, *S* has a fixed point in *P* as Theorem 3 is appropriate to $S, \phi , \psi$ and $(P,d,s,\le )$. $\square$

### *Example 2*

A metric $d:P \rightarrow P$, where $P=\{0, 1, \frac{1}{2},\frac{1}{3},\frac{1}{4},........\frac{1}{n},.....\}$ with usual order $\le$ is defined as follows

$$\begin{aligned} \begin{aligned} d(\nu ,\xi )= {\left\{ \begin{array}{ll} &{} ~0 ~~~~~~~~~,~~ if~ \nu =\xi \\ &{} ~1 ~~~~~~~~~,~~ if~ \nu \ne \xi \in \{0,1\} \\ &{} ~|\nu -\xi |~~~,~~ if~ \nu ,\xi \in \left\{ 0, \frac{1}{2n},\frac{1}{2m}: n \ne m \ge 1\right\} \\ &{} ~5 ~~~~~~~~~~,~~ otherwise. \end{array}\right. } \end{aligned} \end{aligned}$$

A map $S: P \rightarrow P$ be such that $S0=0, S\frac{1}{n}=\frac{1}{9n}$ for all $n\ge 1$ and let $\phi (t)=t$, $\psi (t)=\frac{3t}{4}$ for $t \in [0,+\infty )$. Then *S* has a fixed point in *P*.

### *Proof*

It’s obvious that for $s=\frac{9}{4}$, $(P,d,s,\le )$ is a complete partially ordered *b*-metric space and also by definition, *d* is discontinuous *b*-metric space. Now, for $\nu ,\xi \in P$ with $\nu <\xi$ then consider the following cases:

**Case 1** If $\nu =0$ and $\xi =\frac{1}{n}$, $n \ge 1$, then $d(S\nu ,S\xi )=d(0,\frac{1}{9n})=\frac{1}{9n}$ and $M(\nu ,\xi )=\frac{1}{n}$ or $M(\upsilon ,\xi )= \{1,5\}$. Therefore, we have

$$\begin{aligned} \phi \left( \frac{9}{4}d(S\nu ,S\xi )\right) \le \frac{M(\nu ,\xi )}{4} =\phi (M(\nu ,\xi ))-\psi (M(\nu ,\xi )). \end{aligned}$$

**Case 2** If $\nu =\frac{1}{m}$ and $\xi =\frac{1}{n}$ with $m>n\ge 1$, then

$$\begin{aligned} d(S\nu ,S\xi )=d\left( \frac{1}{9m},\frac{1}{9n}\right) ~\text {and}~ M(\nu ,\xi )\ge \frac{1}{n}-\frac{1}{m}~ \text {or}~ M(\upsilon ,\xi )=5. \end{aligned}$$

Therefore,

$$\begin{aligned} \phi \left( \frac{9}{4}d(S\nu ,S\xi )\right) \le \frac{M(\nu ,\xi )}{4} =\phi (M(\nu ,\xi ))-\psi (M(\nu ,\xi )). \end{aligned}$$

Hence, condition (3) of Theorem 3 and remaining assumptions are satisfied. Thus, *S* has a fixed point in *P*. $\square$

## Limitations

In this manuscript, we obtained a fixed point for generalized $(\phi , \psi )$-weak contraction mapping in a complete partially ordered *b*-metric space. We establish that this mapping has a unique fixed point under ordered relation in a space. Also, we have investigated a common fixed point and a coincidence point for mappings satisfying generalized $(\phi , \psi )$-weak contraction condition in the same space. These results generalize and extended some well known results in the literature. At the end some illustrations are given to support our results.

## Data Availability

Not applicable.
